# Lectin Activity in Commonly Consumed Plant-Based Foods: Calling for Method Harmonization and Risk Assessment

**DOI:** 10.3390/foods10112796

**Published:** 2021-11-13

**Authors:** Anežka Adamcová, Kristian Holst Laursen, Nicolai Zederkopff Ballin

**Affiliations:** 1 Department of Analytical Chemistry, Faculty of Pharmacy in Hradec Králové, Charles University, Heyrovského 1203, 500 05 Hradec Králové, Czech Republic; adamcoa1@faf.cuni.cz; 2 Plant Nutrients and Food Quality Research Group, Plant and Soil Science Section and Copenhagen Plant Science Centre, Department of Plant and Environmental Sciences, Faculty of Science, University of Copenhagen, Thorvaldsensvej 40, 1871 Frederiksberg C, Denmark; holst@plen.ku.dk; 3 Danish Veterinary and Food Administration, Soendervang 4, 4100 Ringsted, Denmark

**Keywords:** active lectins, disease, hemagglutination, lectins, legumes, plant-based foods, risk assessment

## Abstract

Lectins are ubiquitous proteins characterized through their ability to bind different types of carbohydrates. It is well known that active lectins from insufficiently prepared legumes can cause adverse human health effects. The objective of this study was to determine the activity of lectins in samples across plant families representing commercially available edible plants, and the feasibility of inactivating lectins through soaking and boiling. Lectins were extracted from the plant families Adoxaceae, Amaranthaceae, Cannabaceae, Fabaceae, Gramineae, Lamiaceae, Linaceae, Pedaliaceae, and Solanaceae. A hemagglutination assay based on non-treated or trypsin treated rabbit erythrocytes was used to measure the lectin activity. The results showed the highest lectin activity in species from the Fabaceae family and demonstrated that soaking and boiling have an effect on the levels of active lectins. This is the first large study that combines lectin activity obtained from two different assays with raw and processed edible plants. In addition, we examined the current risk assessment, and regulations necessary for an adequate official reporting of results. We encourage the scientific community to further explore this field and agree on harmonized methods for analysis and interpretation, and hope that our methodology can initiate this development.

## 1. Introduction

### 1.1. Plant Lectins and Human Health

Lectins are carbohydrate binding proteins widely distributed in living organisms. In the plant kingdom, lectins are often called phytohemagglutinins (PHA) [1]. Legumes, such as beans and peas, are especially known to be lectin rich. Several studies have found a correlation between legume intake and a lower risk of cardiovascular mortality [2], lower body weights [3], and longevity [4]. However, plant-based food consumption can also be associated with health risks [5]. For example, in legumes, the anti-nutritional components are enzyme inhibitors, tannins, phytates, and lectins. In the food industry, innovative technologies are used to expand the use of legumes and other high-protein plants in food products to partially or totally replace cereals [6], and dietary intake of active lectins might therefore increase in the future [7].

The effects of lectin consumption are mainly monitored in laboratory animals [8]. They can be lethal [9], toxic [10], or even beneficial [11]. One common classification of lectins is based on their affinity towards carbohydrate moieties (Table 1).

As a result of different lectin sugar specificities and their ability to bind different functional compartments of the small intestines, the effect of lectins may vary. Lectins can be extremely resistant to digestive processes and may not be completely degraded in the gastrointestinal tract. Thus, their biological activity is retained during intestinal passage. [15]. Lectins bind to epithelial membrane glycoproteins, such as brush-border membrane enzymes, gangliosides, glycolipids, receptors, secreted mucins, and transport proteins [16]. They are known to disturb the permeability of the intestinal membranes [8], interfere with the absorption of nutrients [17], and lead to extensive damage to the brush border of the proximal small intestine [18]. Moreover, a diet consisting of 1% raw red kidney beans caused deaths in rats within two weeks [9]. 

The acute toxicity in humans caused by active lectins is known from reported incidents of accidental poisoning. Adverse symptoms generally occur within 1–3 h after consumption of insufficiently prepared beans and include nausea, vomiting, diarrhea, and occasional abdominal pain [19]. In the United Kingdom, 50 health incidents associated with red kidney beans were reported between the years 1976 and 1989 [20]. In the Czech Republic, consumption of raw unripe seeds and pods of French beans (*Phaseolus vulgaris*), and runner beans (*Phaseolus coccineus*) were reported to cause acute poisoning, and 13 children required hospitalization between 1996 and 2001 [21]. In Switzerland, species from the genera of *Phaseolus*, *Sambucus*, and *Solanum* were shown to be responsible for severe poisoning between 1995 and 2009 [22]; however, lectins were not identified to be the causal substance. In Japan, more than 1000 cases were linked with consumption of powder roasted white kidney beans in 2006 [23]. In China, 124 health incidents, affecting 7526 individuals, related to fresh kidney beans, were reported between 2004 and 2013 [10]. In Denmark, one known health incident affecting 69 individuals was related to consumption of insufficiently prepared green beans, *Phaseolus coccineus*, in 2013 [24]. To avoid or limit these outbreaks, several measures are important. 

### 1.2. Analysis of Active Lectins

The quantitative methods used to determine the carbohydrate-binding activity of lectins include affinity chromatography [25], immunoenzymatic assay [26], spectrophoto-metry [27], and hemagglutination [20]. However, while a hemagglutination assay could seem a primitive method, it is the most widely used technique; it is cheap, quick, and relatively simple to perform. The technique is semi-quantitative, as active lectins agglutinate erythrocytes through binding of carbohydrate moieties on their surface. In general, the hemagglutination assay is commonly performed by adding a sample solution to an erythrocyte suspension in a microtiter plate. Both human [28] and animal [10] erythrocytes can be used. Compared to human blood, rabbit blood is easily available, cheap, has a lower risk of transmitting infectious diseases, and is therefore more applicable to routine laboratory practice. If a standardized assay and reference material (blood type) is used, the results could be compared between laboratories. Erythrocytes can be treated with trypsin, which will render a broader range of lectins to elicit a positive hemagglutination result [29]. If active lectins are present, agglutinated erythrocytes will spread out and form a diffuse film that will cover the entire surface of the bottom of the well. Non-active lectins will form a sediment of erythrocytes. The results can be observed with the naked eye or with the use of a microscope [30]. A negative result does not necessarily mean that active lectins are not present. For example, some active lectins react only with erythrocytes from specific animal species, if the lectin activity can be masked by the presence of excess free sugars, or by compounds that lyse the erythrocytes [8].

### 1.3. Current Risk Assesment

A guide to preparation of beans, including soaking and cooking times, must be thoroughly communicated to the public. In addition, a standardized assay must be in place to establish the link between lectin activity and adverse health effects but also to monitor lectin activity in food products. The World Health Organization (WHO) recommends that dried beans are soaked for at least 12 h and then boiled vigorously for at least 10 min in water [31]. The U.S. Food and Drug Administration (FDA) recommend soaking in water for 5 h followed by 30 min of cooking [32]. Depending on the type of bean, the Danish Veterinary and Food Administration (DVFA) recommend 10–12 h of soaking followed by 30–60 min of boiling [33]. We have not succeeded in retrieving official national recommendations from other countries. 

The expected increase in plant-based foods is followed by an urgent requirement for information about acute and long-term health consequences. Unfortunately, this information is hindered by the lack of standardized assays, and the scientific knowledge gap in how to characterize the individual lectins and establish their toxicity. The current procedure, for instance, that the DVFA follows, exemplifies the problem we face with this limited knowledge [33]. The DVFA receives samples in cases of suspected outbreaks of lectin poisoning. In the case of kidney beans with a strong record for disease outbreaks, we confidently report positive lectin activity findings followed with a statement that the activity may cause disease and that a treatment to fully inactivate the lectins must be performed. Lectins from other sources are less well studied, leaving us with insufficient knowledge to link outbreaks with a lectin activity finding. In these cases, we report that the lectin activity might cause disease, and that we advise a treatment to inactivate the lectins. However, stronger evidence for a link between lectin activity and disease outbreak is needed to force a change of production procedure, enact punitive measures, or on the other hand, exculpate the lectins from doing harm. Furthermore, the DVFA informs consumers about possible health risks, but with the current knowledge gap in lectin activity and toxicity across plant families, we are ill equipped for the expected increase in plant-based diets and products. This study is intended to add knowledge to the scientific community through an investigation of the lectin activity in legumes and other plant-based foods suspected to contain lectins. Samples from different plant families were soaked and boiled to investigate if these procedures could inactivate lectins. The obtained results are compared and discussed alongside their potential health impacts. 

In summary, the main aims are: (i) to evaluate the content of active lectins in commonly consumed plant foods; (ii) to examine the current risk assessment; and (iii) to propose a harmonized method to examine suspected breakouts.

## 2. Materials and Methods

### 2.1. Samples

Edamame beans, horse beans, ripe and unripe elderberries were self-harvested in Denmark in August 2020. All other samples were purchased from local supermarkets during the fall of 2020. Bell peppers (green, red, yellow), eggplant, green beans (broad, haricot vert), pea leaves, potatoes, sugar peas, sugar snaps, and tomatoes were purchased fresh. Barley, beans (adzukki, borlotti, black, fava, kidney, lima, mung, pinto, rashti, urid, white), chickpeas (brown, yellow), cowpeas, quinoa (black, red, white), lentils (beluga, brown, green, green le Puy, red), rice, seeds (chia, hemp, linseed, nigella, sesame), soybeans, and wheat were purchased dried. Photos of all samples are presented in Picture S1. Latin names representing the common English names of samples were retrieved from online databases and used throughout the manuscript [34,35,36].

### 2.2. Preparation of Samples, Controls, and Commercially Available Lectins

All samples (except of pea leaves) were split into two portions, A and B. “A” samples represent material without subsequent preparation. “B” samples represent material after processing such as soaking and/or boiling (Table 2). Preparation of most samples was conducted according to recommendation from DVFA [33]. When no explicit information about preparation of beans and lentils was available, a comparison in size was used to up or down scale the soaking and boiling times. In respect to vegetables, we chose a preparation that could resemble a standard cooking procedure. 

All unprocessed “A” and processed “B” materials in a minimum weight of 10 g were freeze-dried to obtain the same basis for lectin activity comparison. After freezing at −80 °C, a drying procedure was followed for 48 h in a freeze drier (Scanvac CoolSafe, LaboGene, Lillerød, Denmark). Homogenization was performed with zirconium balls and a shaker mill (model SO-40a, Fluid Management, Holland). Samples of unprocessed barley, beluga lentils, brown lentils, chickpeas (brown, yellow), green lentils, green lentils le Puy, fava beans, lima beans, red lentils, rice, soybeans, wheat, and white beans were further milled using a mixer (Retsch GM200 Grindmixer) at 8500 rpm for 40 s to reach a flour-like fine powder. 

For the hemagglutination assays, organic kidney beans purchased at a local supermarket in 2010 were used as positive and negative controls. For the negative control, kidney beans were soaked for 10 h and then cooked for 30 min. The positive control consisted of kidney beans that were neither soaked nor boiled. The positive and negative controls were homogenized with the Retsch Grindmixer and frozen in 5 g portions at −20 °C. 

Purified lectins from all species included in this work were not commercially available but lectins from red kidney beans (*Phaseolus vulgaris*), soybeans (*Glycine max*), and tomatoes (*Lycopersicon esculentum*, syn. *Solanum lycopersicum*) were purchased from Sigma–Aldrich, St Louis, MO, USA. These species-specific purified lectins established the limit of detection for these three species. Stock solutions (1 mg/mL) of lectins were prepared in phosphate-buffered saline (PBS; 10% *w*/*v*, pH 7.4, Basingstoke, Hampshire, UK) and stored at 5 °C.

### 2.3. Nitrogen Content Analysis

Nitrogen content was analyzed by Dumas combustion using a Vario Macro Cube elemental analyzer (Elementar Analysensysteme GmbH, Hanau, Germany). Prior to weighing into tin capsules and analysis, all samples were dried at 60 °C for a minimum of 2 h. Data quality and standard deviations were determined from the analysis of standard reference materials on 8 individual samples (*n* = 8) (141d acetanilide, National Institute of Standards and Technology (NIST), Gaithersburg, MD, USA and B2166 Birch leaves, Elemental Microanalysis Ltd., Okehampton, Devon, UK) on one day. The nitrogen to protein conversion factors [37,38] used in this study are shown in Table S1.

### 2.4. Lectin Analysis (Hemagglutination Assay)

#### 2.4.1. Preparation of Erythrocyte Cell Suspension

Rabbit, strain Hsdlf NZW, blood suspension in Alsevers liquid (glucose 20.5 g/L, sodium citrate 8.0 g/L, inorganic salt 4.2 g/L, citric acid 0.55 g/L, pH 6.1 ± 0.2), was purchased from SSI Diagnostica (Hillerød, Denmark). A 10% erythrocyte suspension was prepared by transferring 2 mL of rabbit blood suspension into a 10 mL centrifuge tube before centrifugation at 140× *g* for 5 min at room temperature (rt). The supernatant (approximately 1 mL) was carefully removed with a Pasteur pipette. The erythrocytes were then washed three times with 2 mL of cold PBS buffer and centrifuged at 140× *g* for 8 min at rt. After washing, 9 mL of PBS buffer was added. The 10% erythrocyte suspension was prepared weekly from fresh rabbit blood and stored at 5 °C. Two mL of a 10% erythrocyte suspension and 6 mL of PBS buffer were added in a 10 mL centrifuge tube to produce a 2.5% erythrocyte suspension, which was prepared each day of analysis and stored on ice.

#### 2.4.2. Preparation of Trypsin Treated (TT) Erythrocytes

To 2 mL of the 10% erythrocyte suspension, 6 mL of a 0.05% *w*/*v* trypsin (from porcine pancreas, Sigma-Aldrich, St Louis, MO, USA) PBS buffer solution was added to prepare a 2.5% trypsin-treated (TT) erythrocytes suspension. After incubation at 37 °C for 1 h, three washing steps with 6 mL of PBS buffer was performed. The 2.5% TT erythrocyte suspension was prepared each day of analysis and stored on ice.

#### 2.4.3. Extraction of Lectin Samples and Controls

To a 2.5 g portion of the prepared sample material, 5 g of the positive control, and 5 g of the negative control were added 30 g of PBS buffer in 50 mL centrifuge tubes. The tubes were shaken at 250 rpm for 1 h and stored at 4 °C overnight. The slurry was then centrifuged at 2675× *g* for 5 min at rt (room temperature). Five mL of the supernatant were transferred to new 10 mL tubes and centrifuged, as before mentioned, prior to the preparation of the dilution series. 

#### 2.4.4. Preparation of Dilution Series

The dilution series consisted of a non-diluted extract and then a 1:1 serial dilution with PBS buffer. The solutions were prepared by serial dilution in 1.5 mL centrifuge tubes. The first dilution contained 500 µL of PBS buffer and 500 µL of undiluted extract or lectin stock solution. After a 5 s whirl mix at 2500 rpm, 500 µL was added to 500 µL of PBS buffer to produce the next dilution. Subsequent dilutions were performed in the same manner. Dilutions for the samples and the negative control were 1/2, 1/4, 1/8, 1/16, 1/32, 1/64, 1/128, 1/256, 1/512, 1/1024, and 1/2048. The positive control was further diluted 1/4096, 1/8192, and 1/16,384 times. The positive control used in the TT hemagglutination assay was further diluted 1/32,768, 1/65,536, 1/131,072, and 1/262,144 times. The purified lectin stock solutions (1 mg/mL) were diluted 23 times to reach a 1/8,388,608 dilution. 

#### 2.4.5. Sample Application on Microtiter Plates

The hemagglutination assay was performed in 96 wells U-shaped microtiter plates (Greiner bio-one, Stonehouse, UK) as was described earlier [20]. In short, 50 µL of the undiluted and the diluted extracts or lectins stock dilutions were added to wells in a descending dilution order. The negative control was added to the first row of the plate. The positive control was added to the second row and partly into the third row. The blank control (50 µL of PBS buffer) was added into the third row. Following rows included 50 µL of sample extracts or lectin solution. Finally, 50 µL of a 2.5% erythrocyte or TT erythrocyte suspension (gently inverted prior to use) was added to each well and gently shaken. The plates were incubated at 21 °C in a climate chamber for 1–20 h prior to centrifugation at 140× *g* for 3 min at rt.

#### 2.4.6. Reading and Interpretation of Results

Immediately after centrifugation, the plates were visually evaluated. As a prerequisite for plate quality approval, the blank and dilution series of the negative control had to show no hemagglutination in all wells, and the dilution series of the positive control had to show hemagglutination between 1/512–1/1024 dilutions and in the TT hemagglutination assay between 1/16,348–1/65,536 dilutions. The absence of lectins or their inactive form was confirmed with a pellet appearing as a dot on the bottom of the well. The presence of active lectins was confirmed when erythrocytes were spread out covering the surface of the bottom of the well with no pellet formed. 

#### 2.4.7. Calculation of the Hemagglutination Activity Unit (HAU)

The hemagglutination activity of the lectin was expressed as HAU/g dry material and calculated as the reciprocal of the highest dilution that caused hemagglutination multiplied by the initial dilution factor of the sample. For simplicity, hemagglutination activity will be described simply as lectin activity in the following. The relative standard deviation (RSD) was calculated from the positive control analyzed on 12 individual days (*n* = 12). The RSD was used to calculate the standard deviation (SD) for individual samples. An example is presented in the results and discussion section.

## 3. Results and Discussion

### 3.1. Determination of the Limit of Detection and Assay Uncertainty

A positive lectin activity was observed at dilutions 1/16 for *Phaseolus vulgaris*, 1/4096 for *Glycine max*, and 1/1024 for *Lycopersicon esculentum*. The limit of detection was 0.1 mg/mL for *Phaseolus vulgaris*, 0.2 µg/mL for *Glycine max*, and 1.0 µg/mL for *Lycopersicon esculentum* with an initial lectin stock solution of 1 mg/mL. These results show that the ability to agglutinate erythrocytes varies with individual lectins. This means that the same concentration of different lectins will result in vastly different activity levels.

Based on 12 determinations (10 × 1024 HAU/g and 2 × 512 HAU/g) of the positive control on consecutive days, an average of 939 HAU/g with an SD of 199 HAU/g were determined and used to calculate the RSD to 21.2%. This RSD was used to calculate the SD for the individual samples as shown in the following example with beluga lentils. The lectin activity of beluga lentils was 1664 HAU/g, which correspond to an SD of 353 HAU/g (21.2% of 1664 HAU/g). Standard deviations of individual samples are presented in Table 3 and in Figure S1.

**Figure 1:**
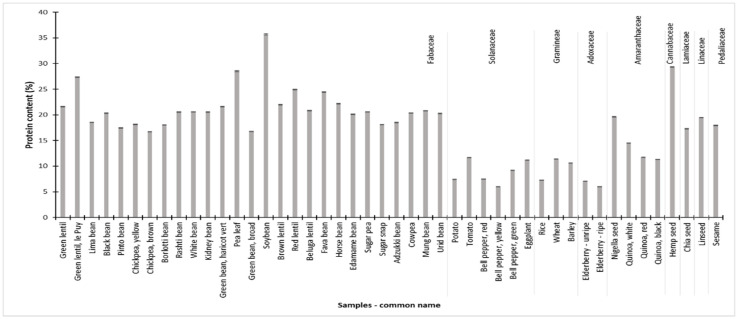
Protein content in unprocessed samples grouped in plant families. Protein content was determined through conversion of nitrogen contents (see Section 2 for methodology). Each bar is the result of one determination (*n* = 1) ± standard deviation (SD). SD was calculated from the positive control, which was analyzed eight times under reproducible conditions. For tabulated results and SD values see Table S1.

Based on eight determinations (10.39, 10.32, 10.31, 10.27, 10.28, 10.24, 10.28, and 10.28% nitrogen) of a standard reference material (acetanilide) within one day, the SD of the nitrogen content was 0.05%, which was converted to a protein content (conversion factor of 5.3, 5.4, or 5.6 depending on plant species) of 0.27–0.28%. These values were used to calculate the SD for each sample following the examples described above with beluga lentils, shown in Figure 1 and Table S1.

### 3.2. Blood Pellet Shape

**Figure 2:**
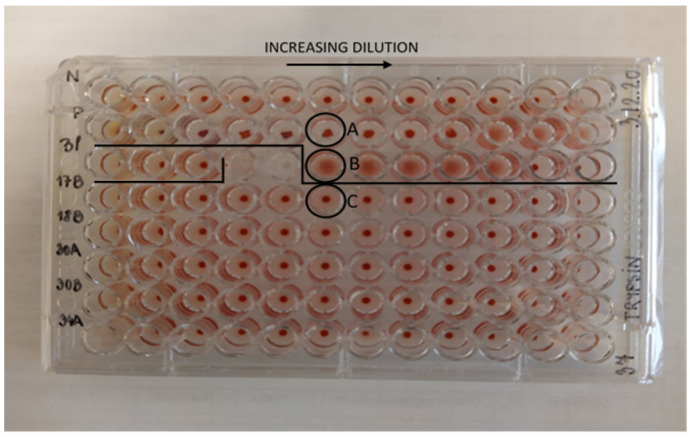
A representative plate of a hemagglutination assay. (N) negative control, (P) positive control, (Bl) blank, numbers (17, 18, 30, 34) represent samples. (A) Shows an irregular shaped pellet from the positive control, (B) shows hemagglutination in the positive control, and (C) shows a lack of hemagglutination in a sample.

Upon approval of plate quality, a differently displayed shape in the wells with a high active lectin concentration was observed. At first glance, this could resemble the regular shaped blood pellet formed at the bottom of wells with no hemagglutination (Figure 2). This irregular shape was observed for many of the unprocessed samples at high lectin concentration, and should be interpreted as an active lectin concentration exceeding the concentrations at which the regular agglutination is possible. However, this is easily distinguished from the regular shaped blood pellet observed in wells with no agglutination, and no ambiguous evaluation was experienced. To compare the irregular shape with agglutination and a lack of agglutination, a microscope was used for magnification (Picture S2).

### 3.3. Non-Trypsin Treated (NTT) Hemagglutination Assay

Table 3 shows lectin activity results in all samples. For graphic expression of lectin activity results of all unprocessed samples, see Figure S1. After plate quality approval, results from individual samples were obtained through the NTT hemagglutination assay. 

From the Fabaceae family, samples of beans (black, borlotti, fava, kidney, lima, pinto, rashti, white), fresh green beans (broad, edamame, haricot vert, horse), lentils (beluga, brown, red), soybeans, sugar peas, and sugar snaps had lectin activities of 208–26,526 HAU/g. The highest lectin activity was found in lima beans and the lowest in sugar snaps. In vivo studies have previously shown samples of unprocessed lima beans as toxic. Lima beans did not support proper growth of rats although the animals survived the 10 days experimental period [39]. Others reported lima beans as highly toxic [8], however no adverse health incidents have been reported. Unprocessed fresh broad green beans, red and white kidney beans are classed as highly toxic [8,39] and there are many known acute health incidents [10,20,21,23,24]. Pea leaves, sugar peas, and sugar snaps are consumed fresh without soaking and heat treatment, and the lectins are therefore consumed in an active form, but classified as non-toxic [39] or slightly toxic [8].

From the Solanaceae family, samples of bell peppers (red, yellow), potatoes, and tomatoes had lectin activities of 26–826 HAU/g. The presence of lectins in this family is well established [28,40]. The highest activity among samples in this family was determined in potatoes, which is consistent with another study [28]. Active lectins were detected in tomatoes and their presence was not removed by boiling for 5 min. Bell peppers and tomatoes are often consumed raw and lectins are therefore consumed in the active form, but evident from the frequent consumption of these commodities, no general acute adverse health effects have been reported. Potato and tomato lectins are known to be non-toxic [8].

From the Adoxaceae family, samples of elderberries had lectin activities of 52–104 HAU/g. Active lectins were found in ripe and unripe elderberries. Lectins in unripe elderberries were not removed after boiling for 15 min, which has also been previously demonstrated [41]. This in vitro study showed that heat treatment of elderberries sensitized lectins to the pepsin and therefore reduced allergy-related risks. Peumans and Van Damme [8] indicated harmful effect in raw elderberries and possible harmful effect in processed elderberries. Our results do not rule out the possibility that lectins could be responsible for the adverse effect of elderberries.

From the Amaranthaceae family, samples of quinoa (black, red, white) and nigella seeds had lectin activities of 24–104 HAU/g. The lectin activity in nigella seeds was not removed after 5 min of boiling. However, even if nigella seeds contain lectins problematic for human health, it is assumed that the seeds are currently consumed in quantities that do not pose a problem. Another study also showed a low level of active lectins in quinoa [42].

The NTT hemagglutination assay did not show agglutination in samples from the families of Cannabaceae (hemp seeds), Fabaceae (adzukki beans, brown and yellow chickpeas, cowpeas, green lentils, green lentils le Puy, mung beans, urid beans), Gramineae (barley, rice, wheat), Lamiaceae (chia seeds), Linaceae (linseeds), Pedaliaceae (sesame), and Solanaceae (eggplant, green bell pepper). All the above mentioned samples were subjected to the TT hemagglutination assay, which has successfully shown active lectins in chickpeas [29]. In the case of chickpeas, no lectin activity in red blood cell from humans, rats, rabbits, or monkeys without trypsin treated blood was reported [43], whereas the activity in cow erythrocytes was observed [44].

### 3.4. Trypsin Treated (TT) Hemagglutination Assay

Table 3 shows lectin activity results in all samples. For a graphic presentation of lectin activity results of all unprocessed samples, see Figure S1. After plate quality approval, results from individual samples were obtained through the TT hemagglutination assay.

In the TT hemagglutination assay, green lentils and green lentils le Puy had lectin activity of 1,703,936–3,407,872 HAU/g, which represents the highest activity in this study. This is in line with another study [45]. The TT hemagglutination assay also detected active lectins in both types of chickpeas (13,312 HAU/g) and rice (208 HAU/g). Contrary to other samples, brown and yellow chickpeas also showed a high content of active lectins at 13,312 and 6656 HAU/g before and after processing, respectively. There are conflicting reports on their toxicity. For instance, one study reported that lectins from lentils have a slight oral toxicity and may be harmful to humans if consumed raw [8]. However, other authors have found that lectins from chickpeas and lentils are non-toxic [39,46].

To investigate the unexpected results in chickpea samples a second portion were analyzed, and identical results were obtained. We do not have an explanation for the apparent heat resistance observed for lectins from chickpeas. Our curiosity prompted us to purchase two different brands of falafel (composed mostly of chickpeas) and heat them according to the package description prior to analyses. These results confirmed the high lectin activity found in chickpeas. The fact that this is an everyday commodity for many consumers exemplifies the lack of a clear correlation between lectin activities obtained in the TT hemagglutination assay and acute adverse health effects. 

None of the assays detected active lectins in adzukki beans, barley, chia seeds, cowpeas, eggplant, green bell pepper, hemp seeds, linseeds, mung beans, sesame, urid beans, or wheat. The lack of lectin activity in samples of adzukki beans, cowpeas, mung beans, and urid beans is in contrast to literature data that showed hemagglutination with TT rabbit erythrocytes [14]. These contrary results could be explained by the usage of blood from different rabbit strains, but this information is often not disclosed. Furthermore, it cannot be excluded that sample impurities (biomolecules) could affect agglutination both *in vivo* and *in vitro*. This could be the subject of further research.

### 3.5. The Effect of Preparation

All samples, except for pea leaves, were prepared according to the procedure described in Table 2, and analyzed with the NTT or TT hemagglutination assay depending on where lectin activity was observed for the unprepared portion of the sample. Except for elderberries, chickpeas, nigella seeds, and tomatoes, all samples showed a total inactivation of lectins after preparation. This is in line with the literature showing a loss or reduction of adverse health effects after soaking and/or boiling [10,41,47]. Unfortunately, clear guidelines for the soaking and boiling preparation of legumes are missing. The WHO suggest to soak the beans for at least 12 h and subsequently to boil them for 10 min but do not further describe how to boil (slow vs. fast cooking). The handbook “Bad Bug Book—Foodborne Pathogenic Microorganisms and Natural Toxins” published by the FDA includes a study by Bender and Reaidi [48] that found a complete destruction of the toxin after 10 min (100 °C) of boiling, but then further adds that consumers should soak the beans for at least 5 h and boil them for at least 30 min to ensure that lectins are completely destroyed [48]. They also encourage the avoidance of using slow cookers that usually do not reach adequate temperature for destruction of the lectin [32]. This is in line with Safefood (the public authority responsible for raising consumer awareness of issues relating to food safety and healthy eating across both the Republic of Ireland and Northern Ireland), that confirm slow cooking does not destroy lectins in raw beans because the temperature is not high enough [49]. The Danish Veterinary and Food Administration recommend 10–12 h of soaking and 30–60 min of boiling depending on the specific bean types. We have demonstrated that the DVFA suggested procedure for soaking and boiling preparation is sufficient for inactivation of active lectins [33]. It must be mentioned that not only boiling and following cooking are used to process legumes. For instance, extrusion cooking technology can be used to prepare extruded foods with reduction of lectins (and other anti-nutrients) as thoroughly discussed in a recent review by Pasqualone et al. [6].

### 3.6. Evaluation of the Non-Trypsin Treated (NTT) and the Trypsin Treated (TT) Assays

In this study, samples of common plant foods were subjected to the NTT assay, and in cases with no lectin activity, the TT assay was used. It is now obvious that the genera *Glycine, Phaseolus, Pisum*, and *Vicia* can agglutinate NTT erythrocytes. The genera *Cicer* (only one species included in the study, namely *arietinum*) agglutinated only TT erythrocytes, and *Vigna* species did not show any activity. For species within the genus *Lens*, the subspecies green lentils and green lentils le Puy agglutinated TT erythrocytes; however, beluga, brown and red lentils agglutinated NTT erythrocytes. It remains unclear how to determine beforehand which commodities give a response in one or the other assay because a closer look at the species and subspecies (in case of genus *Lens*) reveals no clear picture. We tried to focus on sugar moieties to make a distinction between the agglutination capabilities of different plant species on the NTT and TT hemagglutination assay, but as evident from Table 1, no clear distinction can be made. However, it seems that all species from the genus *Vigna* have lectins that bind galactose. Lectins with a single specificity toward galactose are probably disqualified for lectin activity evaluation through the presented assays. In summary, there is no easy way to determine which samples and plant species should be analyzed with the TT or NTT hemagglutination assays, or which samples cannot be analyzed with these assays. However, the preparation suggested by the DVFA and used in this paper is sufficient to deactivate lectins in beans and lentils, with the exception of chickpeas.

### 3.7. Threshold Limits of Active Lectins in Plant-Based Products

Fresh broad green beans (*Phaseolus coccineus*) and red and white kidney beans have been reported to cause acute symptoms (nausea, vomiting, diarrhea) [20,21,23,24] and are classified as highly toxic [8,39]. According to Pusztai and Palmer [50], the toxic effect of kidney beans is related to lectin content and hence, hemagglutination activity. Laboratory confirmed cases of kidney bean poisons in the United Kingdom reported the concentration of active lectins in these incidents between 3200 and 102,000 HAU/g, and considered the range between 400 HAU/g and 3200 HAU/g as possibly toxic [20]. In the absence of standardized procedure (freeze-dried or not), standardized assays, reference materials, and proficiency tests, we cannot establish a common threshold level. Nonetheless, we can report that lectin activity from plants with a record of adverse health issues might cause disease (depending on the amount of intake) and such beans should be prepared sufficiently to inactivate lectins.

### 3.8. Nitrogen and Protein Content

The nitrogen content of all samples varied from 1.1% to 6.5%. The corresponding protein content ranged from 6.0% (bell peppers and elderberries) to 35.7% (soybeans) as shown in Figure 1, and more elaborated in Table S1. Samples from the Amaranthaceae, Cannabaceae, Fabaceae, Lamiaceae, Linaceae, and Pedaliaceae families generally displayed the highest protein contents. Individual plant species able to conduct biological nitrogen fixation had the highest protein contents in most cases. No apparent correlation (*r* = 0.098) between the protein content and the active lectin concentration was observed across all species, which was also reported elsewhere [28].

## 4. Conclusions

We reported lectin activities with the NTT and the TT hemagglutination assay for several plant products before and after preparation. The Danish national recommendations on soaking and boiling times were tested in this work and showed a complete inactivation of lectins in beans. Despite the fact that we did not assay the toxicity, we evaluated the possible link with NTT assay results and adverse effects in humans. A high lectin activity level is not necessarily linked to oral toxicity in humans, but the specific assay might provide a first indication. It was only in the NTT hemagglutination assay that we observed species with lectin activity levels associated with previously reported adverse health effects. 

For the first time, we addressed the apparent lack of official threshold levels for active lectins in food. To establish these levels, we need a risk assessment based on lectin toxicity. A prerequisite for this assessment is toxicity data, which needs standardized assays, reference materials, and proficiency tests to enable valid and comparable results across laboratories. As a starting point, we recommend the NTT assay, freeze-dried samples, rabbit blood from one strain and, centrifugation of microtiter plates. Harmonized official recommendations on how to prepare beans prior to consumption should be communicated to the public based on sound evidence. The food industry and the national and international food authorities and disease control units will benefit greatly from this work. There is no reason why lectins should be treated differently compared to all the non-protein natural toxins that are safety evaluated and regulated in various food laws.

## Figures and Tables

**Table 1:** Sugar specificity of lectins from selected plant species.

Species and Common Plant Name	Sugar Specificity ^†^	References
*Chenopodium quinoa* Willd. (quinoa—black, red, white)	(GlcNAc)_n_	[12]
*Cicer arietinum* L. (chickpea)	Complex	[12]
*Glycine max* L. (soybean)	GalNAc > Gal	[12]
*Hordeum vulgare* L. (barley)	GlcNAc	[12]
*Lens culinaris* L. (beluga lentil, brown lentil, green lentil, green lentil le Puy, red lentil)	Man/Glc	[12]
*Oryza sativa* L. (rice)	GlcNAc	[12]
*Phaseolus lunatus* L. (lima bean)	GalNAc	[8,12]
*Phaseolus vulgaris* L. (black bean, borlotti bean, green bean—haricot vert, kidney bean, rashti bean, white bean)	Complex	[12]
*Pisum sativum* L. (pea)	Man/Glc	[12]
*Sambucus nigra* L.—fruit (elderberry)	Gal/GalNAc	[12]
*Solanum lycopersicum* L. (tomato)	(GlcNAc)_n_	[12]
*Solanum tuberosum* L. (potato)	GalNAc > Gal	[12,13]
*Triticum aestivum* L. (wheat)	GlcNAc	[12]
*Vicia faba* L. (fava bean, horse bean)	Man/Glc	[12]
*Vigna angularis* L. (adzukki bean)	Gal	[13]
*Vigna mungo* L. (urid bean)	Gal	[13]
*Vigna radiata* L. (mung bean)	Gal	[14]
*Vigna unguiculata* L. (cowpea)	Gal	[14]

**^†^** Gal, galactose; GalNAc, N-Acetylgalactosamine; Glc, glucose; GlcNAc, N-Acetylglucosamine; Man, mannose. Species with complex sugar specificity are able to bind a wide range of monosaccharides.

**Table 2:** Common names, species names, geographical origin, and sample preparation.

Family Common Name	Species Name	Geographic Origin	Sample Preparation	
			Soaking	Boiling
Adoxaceae				
Elderberry—ripe	*Sambucus nigra* L.	Denmark	-	15 min
Elderberry—unripe	*Sambucus nigra* L.	Denmark	-	15 min
Amaranthaceae				
Nigella seed	*Nigella sativa* L.	India/Egypt	-	5 min
Quinoa, black	*Chenopodium quinoa* Willd.	Peru, Bolivia	-	15 min
Quinoa, white	*Chenopodium quinoa* Willd.	Unknown	-	20 min
Quinoa, red	*Chenopodium quinoa* Willd.	Peru, Bolivia	-	15 min
Cannabaceae				
Hemp seed	*Cannabis sativa* L.	China	-	5 min
Fabaceae				
Adzukki bean	*Vigna angularis* L.	Unknown	12 h	45 min
Beluga lentil	*Lens culinaris* L.	Turkey	-	20 min
Black bean	*Phaseolus vulgaris* L.	China	12 h	30 min
Borlotti bean	*Phaseolus vulgaris* L.	China	12 h	45 min
Brown lentil	*Lens culinaris* L.	Canada	-	20 min
Chickpea, brown	*Cicer arietinum* L.	India	12 h	1 h
Chickpea, yellow	*Cicer arietinum* L.	EU	12 h	1 h
Cowpea	*Vigna unguiculata* L.	Argentina	12 h	30 min
Edamame bean	*Glycine max* L.	Denmark	-	5 min
Fava bean	*Vicia faba* L.	Unknown	12 h	1 h
Green bean, broad	*Phaseolus coccineus* L.	Spain	-	10 min
Green bean, haricot vert	*Phaseolus vulgaris* L.	Nederland	-	10 min
Green lentil	*Lens culinaris* L.	Turkey	-	20 min
Green lentil, le Puy	*Lens culinaris* L.	Turkey	-	20 min
Horse bean	*Vicia faba* L.	Denmark	-	5 min
Kidney bean	*Phaseolus vulgaris* L.	Unknown	12 h	30 min
Lima bean	*Phaseolus lunatus* L.	Unknown	12 h	30 min
Mung bean	*Vigna radiata* L.	Uzbekistan	12 h	20 min
Pea leaves	*Pisum sativum* L.	Denmark	-	-
Pinto bean	*Phaseolus vulgaris* L.	Unknown	12 h	45 min
Rashti bean	*Phaseolus vulgaris* L.	Unknown	12 h	45 min
Red lentil	*Lens culinaris* L.	Turkey	-	10 min
Soybean	*Glycine max* L.	Canada	12 h	45 min
Sugar pea	*Pisum sativum* L.	Denmark		5 min
Sugar snap	*Pisum sativum* L.	Denmark	-	5 min
Urid bean	*Vigna mungo* L.	Myanmar	12 h	20 min
White bean	*Phaseolus vulgaris* L.	China	12 h	45 min
Gramineae				
Barley	*Hordeum vulgare* L.	Denmark	-	15 min
Rice	*Oryza sativa* L.	Unknown	-	15 min
Wheat	*Triticum aestivum* L.	Denmark	-	15 min
Lamiaceae				
Chia seed	*Salvia hispanica* L.	Paraguay	20 min	-
Linaceae				
Linseed	*Linum usitatissimum* L.	India	-	5 min
Pedaliaceae				
Sesame	*Sesamum indicum* L.	Uganda/India/Pakistan	-	5 min
Solanaceae				
Bell pepper, green	*Capsicum annuum* L.	Nederland	-	5 min
Bell pepper, red	*Capsicum annuum* L.	Nederland	-	5 min
Bell pepper, yellow	*Capsicum annuum* L.	Nederland	-	5 min
Eggplant	*Solanum melongena* L.	Nederland	-	10 min
Potato	*Solanum tuberosum* L.	Denmark	-	20 min
Tomato	*Solanum lycopersicum* L.	Denmark	-	5 min

**Table 3:** Lectin activity (HAU/g) results obtained with the non-trypsin treated and trypsin treated hemagglutination assay using rabbit erythrocytes in raw and processed material.

Family	Raw Material (HAU/g)		Processed Material (HAU/g) ^†^	
Common Name	NTT ± SD	TT ± SD	NTT ± SD	TT ± SD
Adoxaceae				
Elderberry—ripe	52 ± 11	-	ND	-
Elderberry—unripe	104 ± 22	-	26 ± 6	-
Amaranthaceae				
Nigella seed	104 ± 22	-	26 ± 6	-
Quinoa, black	26 ± 6	-	ND	-
Quinoa, white	26 ± 6	-	ND	-
Quinoa, red	26 ± 6	-	ND	-
Cannabaceae				
Hemp seed	ND	ND	ND	ND
Fabaceae				
Adzukki bean	ND	ND	ND	-
Beluga lentil	1664 ± 353	-	ND	-
Black bean	26,429 ± 5603	-	ND	-
Borlotti bean	13,312 ± 2822	-	ND	-
Brown lentil	3328 ± 706	-	ND	-
Chickpea, brown	ND	13,312 ± 2822	ND	6656 ± 1411
Chickpea, yellow	ND	13,312 ± 2822	ND	13,312 ± 2822
Cowpea	ND	ND	ND	-
Edamame bean	416 ± 88	-	ND	-
Fava bean	1658 ± 351	-	ND	-
Green bean, broad	3328 ± 706	-	26 ± 6	-
Green bean, haricot vert	6656 ± 1411	-	ND	-
Green lentil	ND	3,407,872 ± 722,469	ND	ND
Green lentil, le Puy	ND	1,703,936 ± 361,234	ND	ND
Horse bean	1658 ± 351	-	ND	-
Kidney bean	13,214 ± 2828	-	ND	-
Lima bean	26,526 ± 5624	-	ND	-
Mung bean	ND	ND	ND	-
Pea leaf	4901 ± 1039	-	-	-
Pinto bean	13,563 ± 2875	-	ND	-
Rashti bean	13,312 ± 2822	-	ND	-
Red lentil	3328 ± 706	-	ND	-
Soybean	3328 ± 706	-	ND	-
Sugar pea	414 ± 88	-	ND	-
Sugar snap	208 ± 44	-	ND	-
Urid bean	ND	ND	ND	-
White bean	13,263 ± 2812	-	ND	-
Gramineae				
Barley	ND	ND	ND	-
Rice	-	208 ± 44	ND	ND
Wheat	ND	ND	ND	-
Lamiaceae				
Chia seed	ND	ND	ND	ND
Linaceae				
Linseed	ND	ND	ND	ND
Pedaliaceae				
Sesame	ND	ND	ND	ND
Solanaceae				
Bell pepper, green	ND	ND	ND	ND
Bell pepper, red	26 ± 6	-	ND	-
Bell pepper, yellow	26 ± 6	-	ND	-
Eggplant	ND	ND	ND	ND
Potato	826 ± 175	-	ND	-
Tomato	256 ± 54	-	104 ± 22	-

^†^ The preparation of each sample is shown in Table 2. The relative standard deviation (RSD) was calculated from the positive control, which was analyzed 12 times under reproducible conditions. The RSD was used to calculate the SD for individual samples that was analyzed once (*n* = 1). ND, not detected; NTT, non-trypsin treated hemagglutination assay; TT, trypsin treated hemagglutination assay; -, the hemagglutination assay was not performed.

## Data Availability

Not relevant.

## Supplementary Materials

The following are available online at https://www.mdpi.com/article/10.3390/foods10112796/s1, Picture S1—Sample pictures, Table S1—Nitrogen results, Figure S1—Lectin activity results for all unprocessed samples, Picture S2—Differently displayed results.
