# Supplementary material for: Lectin Activity in Commonly Consumed Plant-Based Foods: Calling for Method Harmonization and Risk Assessment
Source: Foods. 2021 Nov 13;10(11):2796. doi: 10.3390/foods10112796 (PMC8618113; doi:10.3390/foods10112796)
Supplement: Supplementary file 1 [file foods-10-02796-s001.zip › Figure S1 - Lectin activity results for all unprocessed samples.pdf]

Figure S1 – Lectin activity results for all unprocessed samples

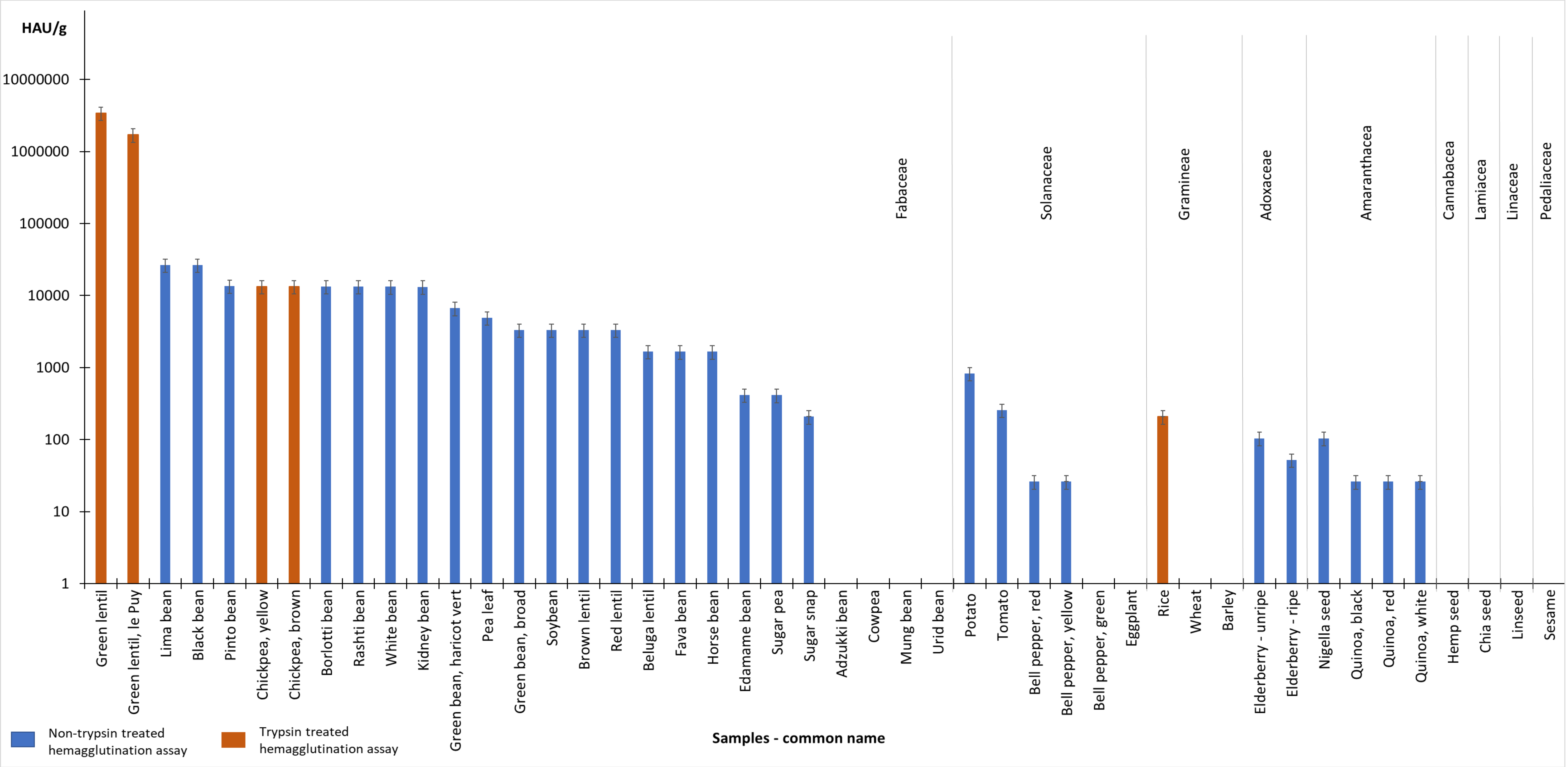

Figure S1. Lectin activity (HAU/g) in unprocessed samples grouped in plant families. The results were measured with the non-trypsin treated and the trypsin treated hemagglutination assay. Within each family, samples are sorted in a descending lectin activity order. Each bar is the result of one determination (n = 1) ± standard deviation (SD). The RSD was calculated from the positive control, which was analysed 12 times under reproducible conditions. The RSD was then used to calculate the SD for individual samples. For tabulated SD values see Table 3.
