# Supplementary material for: Lectin Activity in Commonly Consumed Plant-Based Foods: Calling for Method Harmonization and Risk Assessment
Source: Foods. 2021 Nov 13;10(11):2796. doi: 10.3390/foods10112796 (PMC8618113; doi:10.3390/foods10112796)
Supplement: Supplementary file 1 [file foods-10-02796-s001.zip › Picture S1 - Sample pictures.pdf]

Picture S1 – Sample pictures

Adoxaceae family

Elderberry - ripe
Sambucus nigra L.

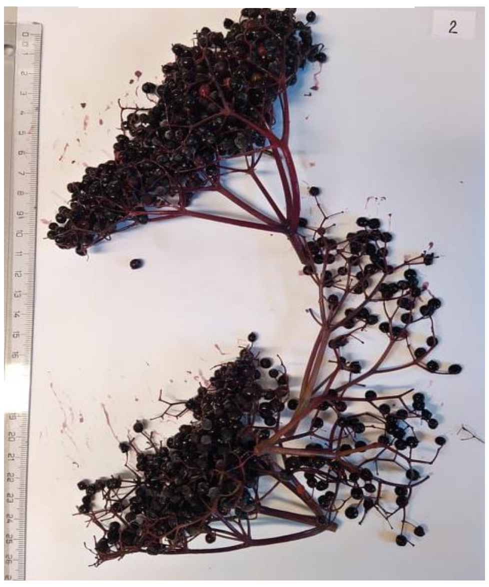

Elderberry - unripe
Sambucus nigra L.

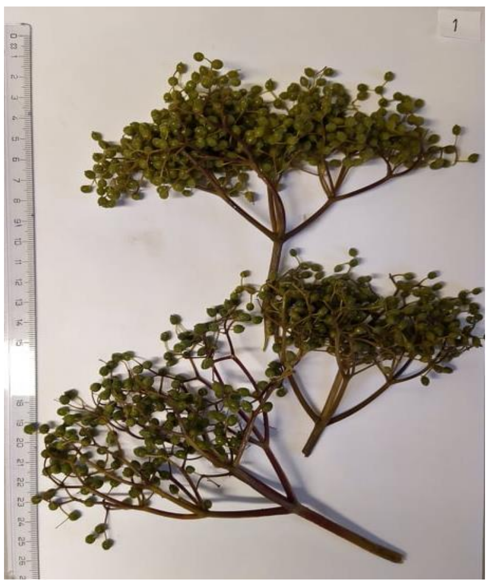

Amaranthaceae family

Nigella seed
Nigella sativa L.

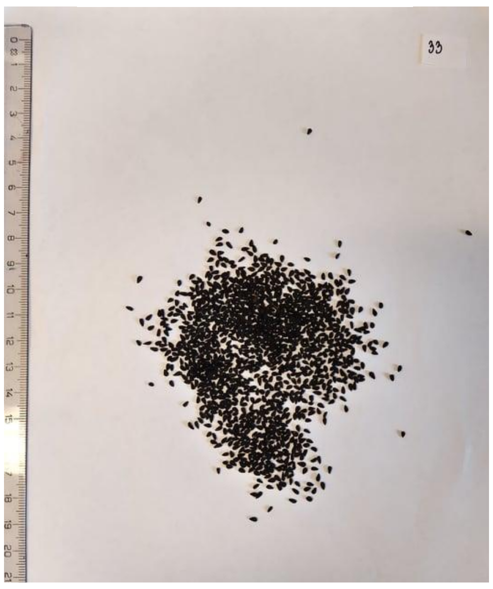

Quinoa, black
Chenopodium quinoa Willd.

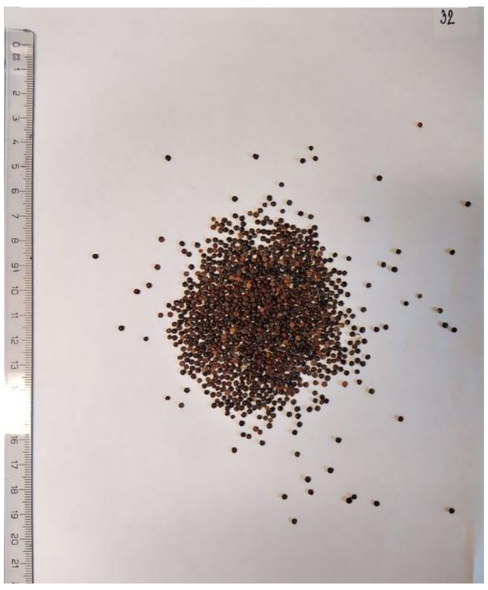

Quinoa, red
Chenopodium quinoa Willd.

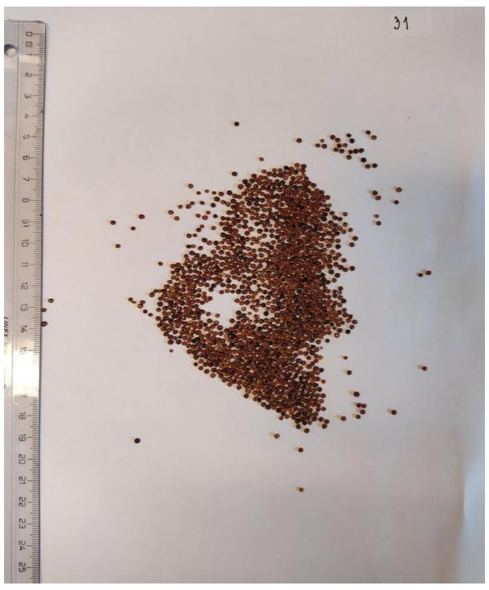

Quinoa, white
Chenopodium quinoa Willd.

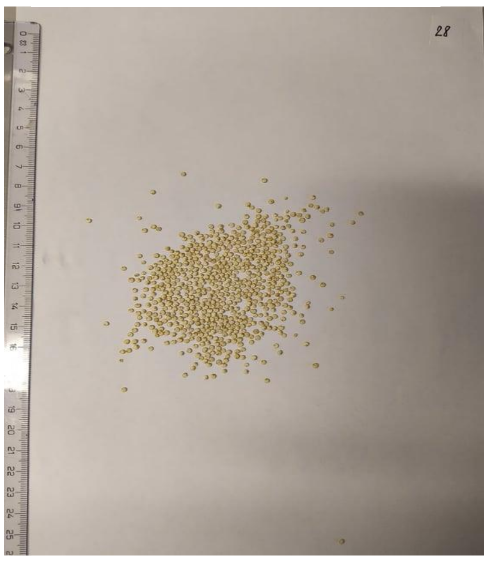

Cannabaceae family

Hemp seed
Cannabis sativa L.

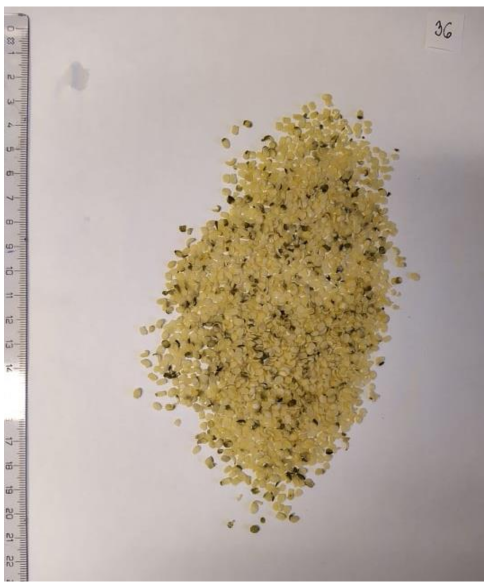

Picture S1 – Sample pictures

Fabaceae family

Adzukki bean
Vigna angularis L.

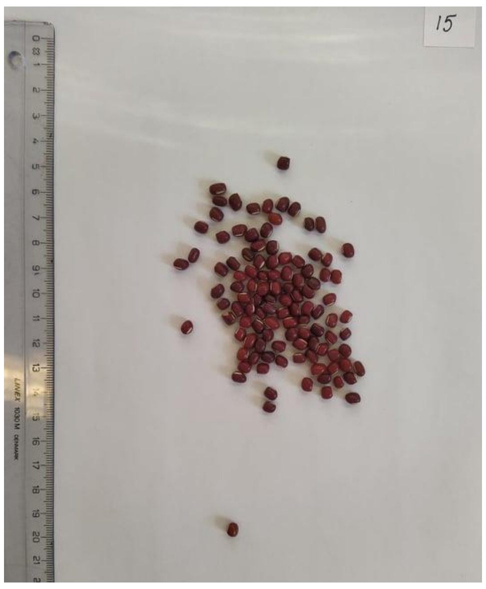

Beluga lentil
Lens culinaris L.

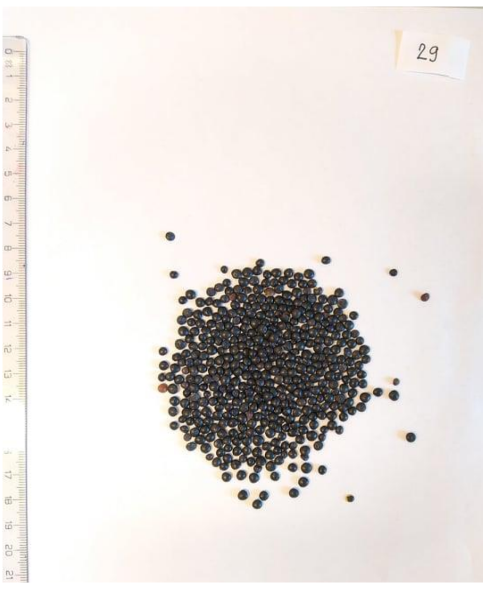

Black bean
Phaseolus vulgaris L.

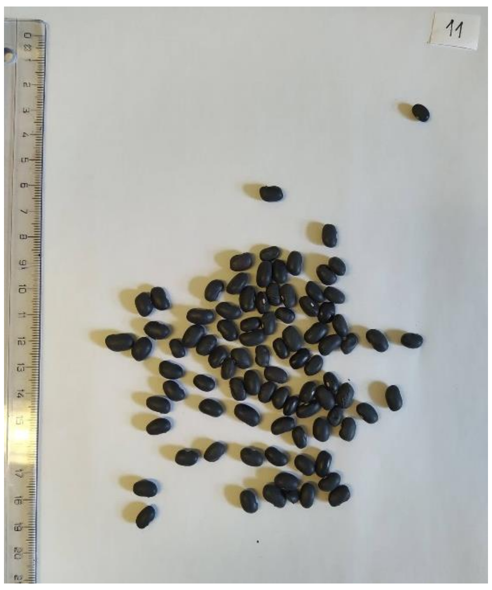

Borlotti bean
Phaseolus vulgaris L.

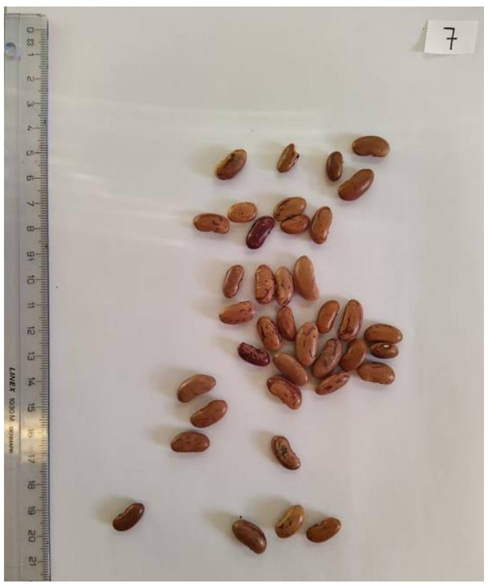

Brown lentil
Lens culinaris L.

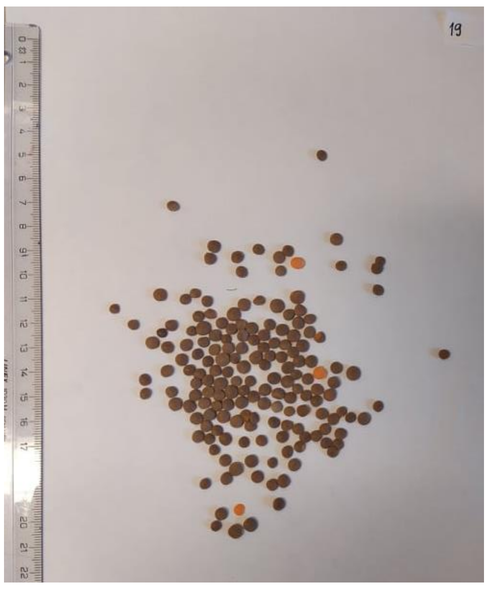

Chickpea, brown
Cicer arietinum L.

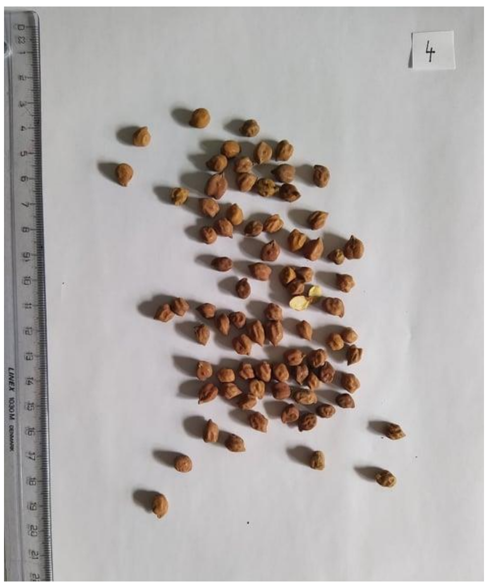

Chickpea, yellow
Cicer arietinum L.

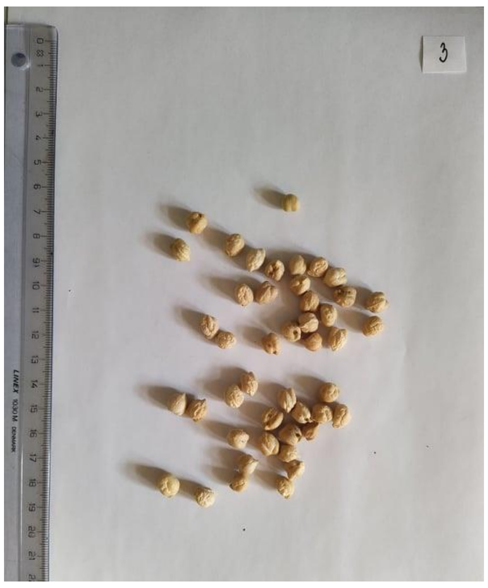

Cowpea
Vigna unguiculata L.

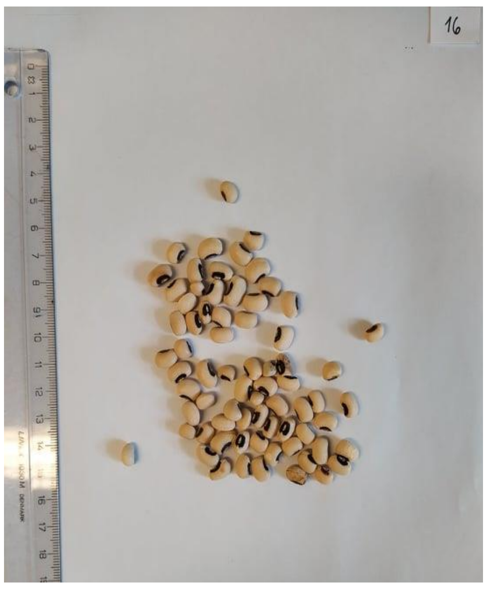

Edamame bean
Glycine max L.

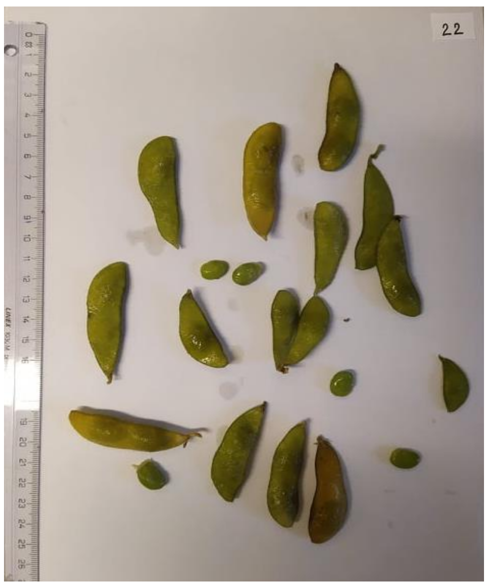

Fava bean
Vicia faba L.

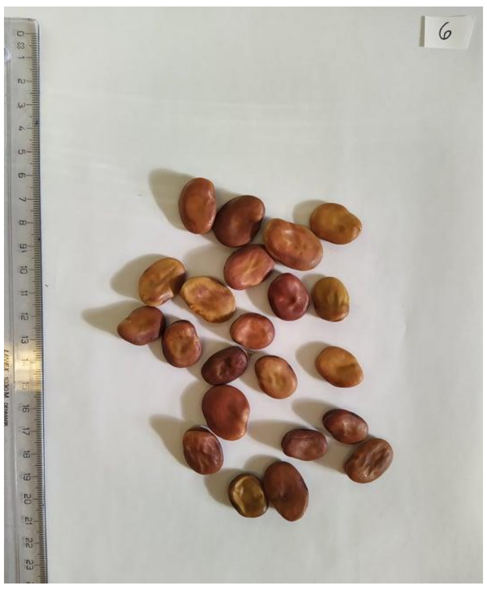

Green bean, broad
Phaseolus coccineus L.*

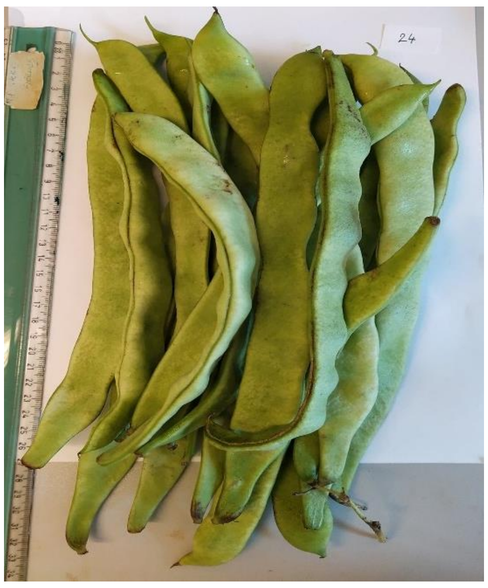

Green bean, haricot vert
Phaseolus vulgaris L.

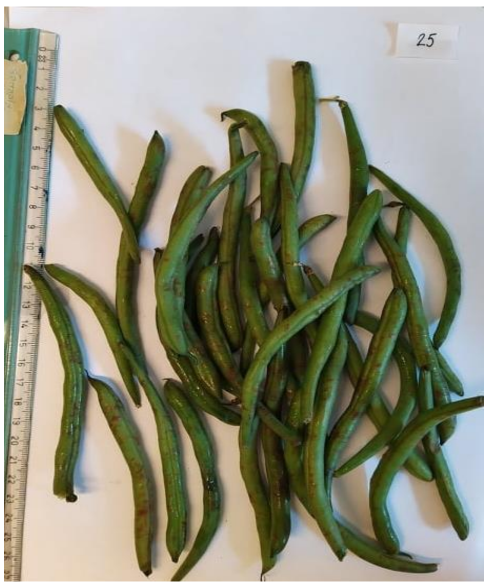

Picture S1 – Sample pictures

Green lentil
Lens culinaris L.

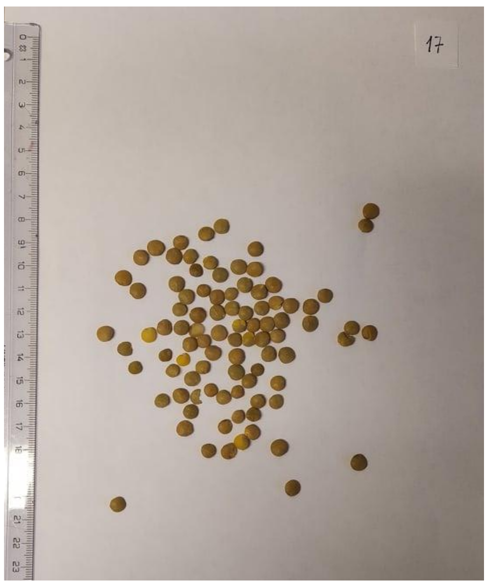

Green lentil, le Puy
Lens culinaris L.

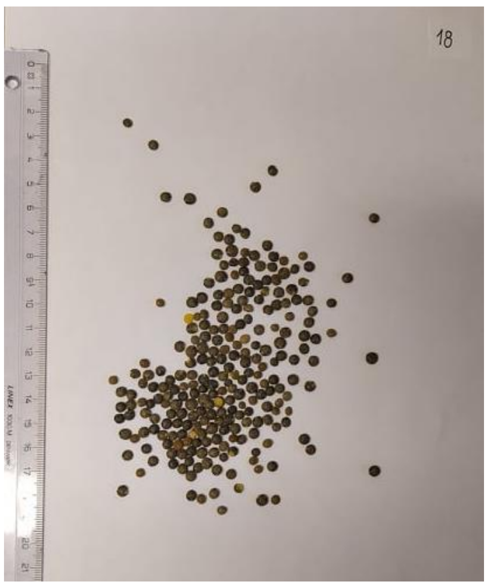

Horse bean
Vicia faba L.

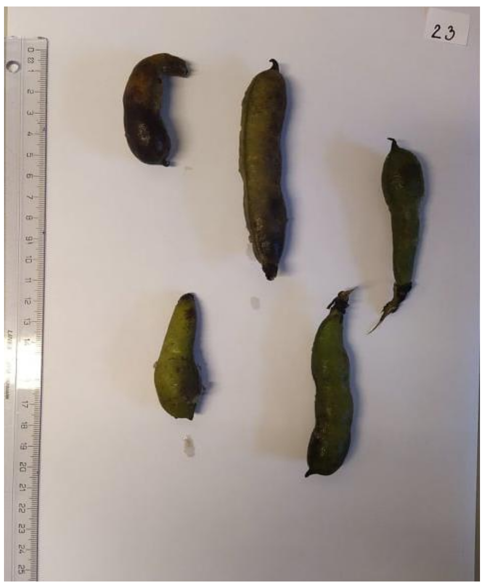

Kidney bean
Phaseolus vulgaris L.

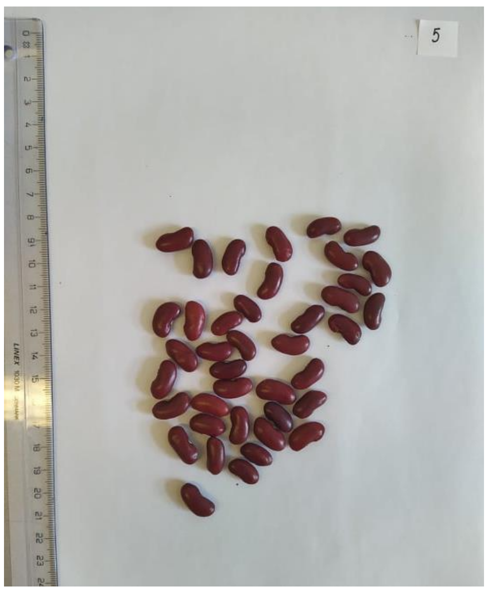

Lima bean
Phaseolus lunatus L.

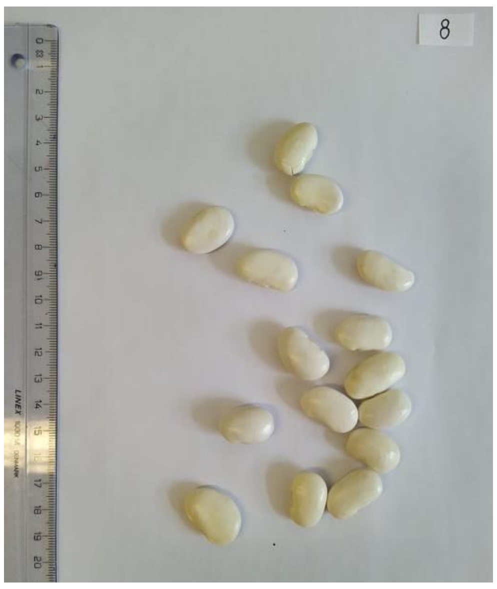

Mung bean
Vigna radiata L.

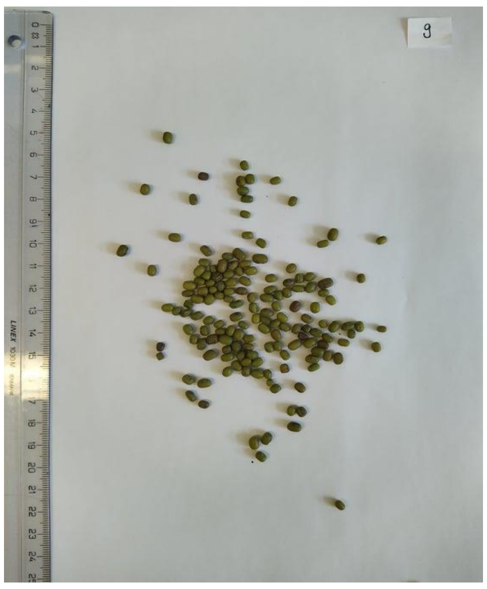

Pea leaf
Pisum sativum L.

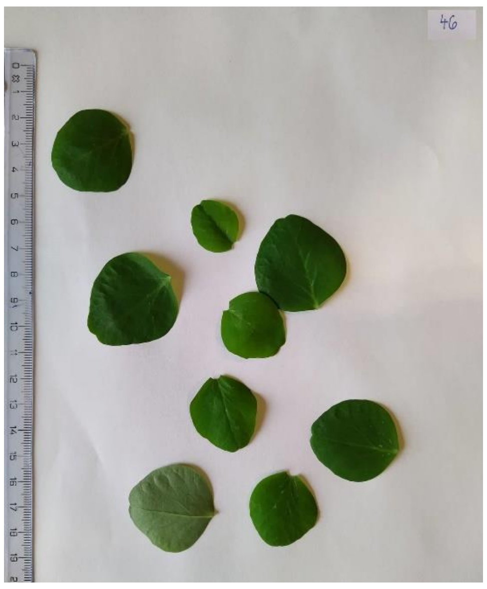

Pinto bean
Phaseolus vulgaris L.

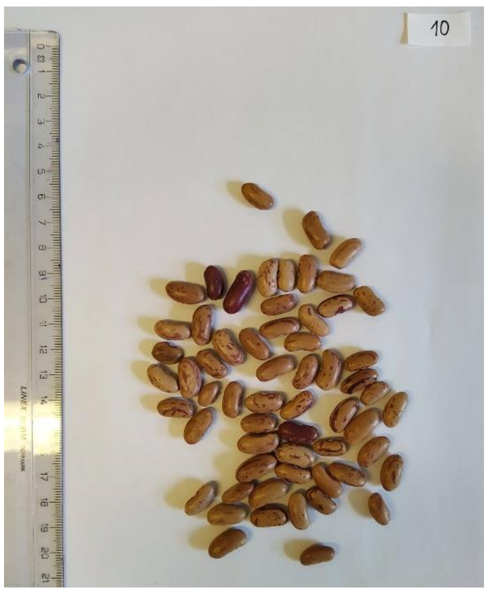

Rashti bean
Phaseolus vulgaris L.

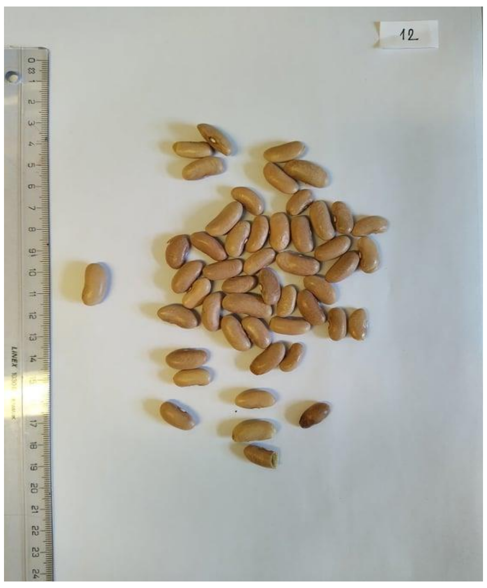

Red lentil
Lens culinaris L.

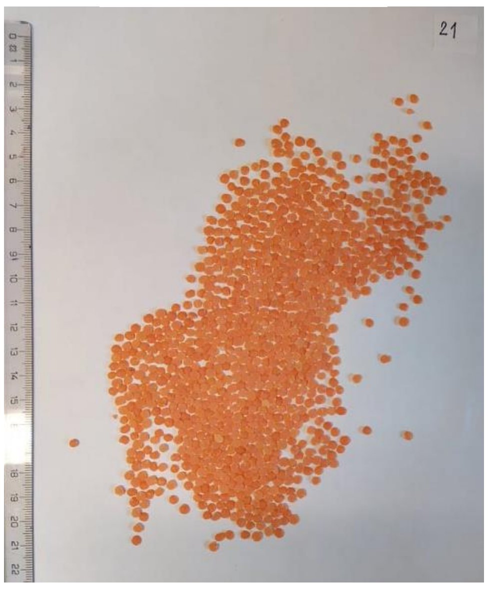

Soybean
Glycine max L.

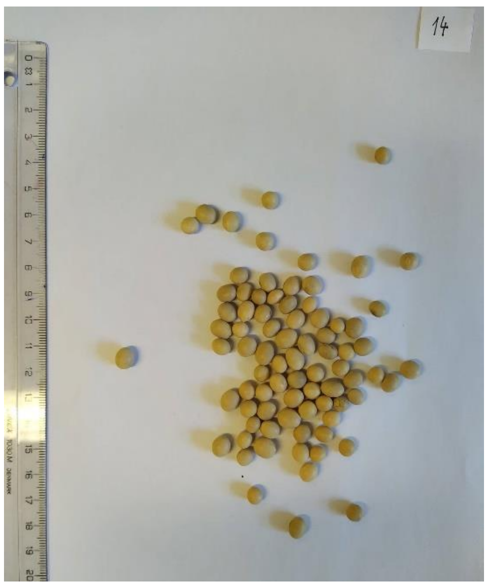

Sugar pea
Pisum sativum L.

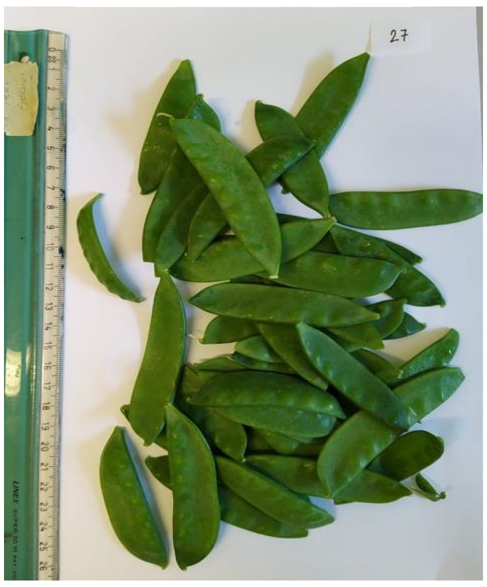

Picture S1 – Sample pictures

Sugar snap
Pisum sativum L.

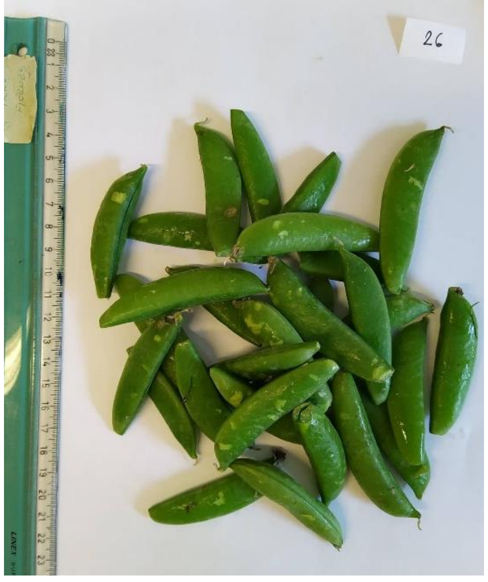

Urid bean
Vigna mungo L.

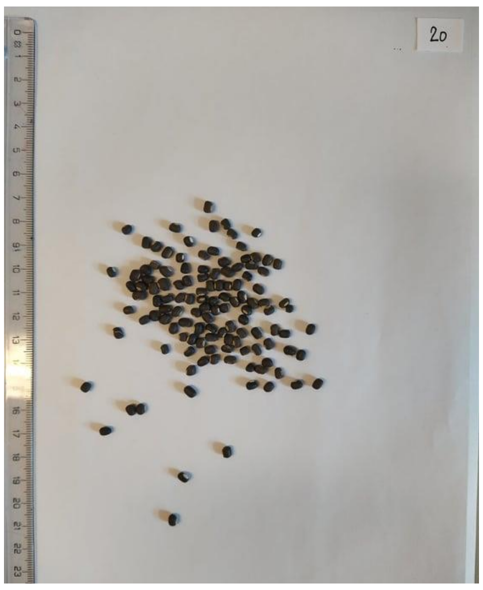

White bean
Phaseolus vulgaris L.

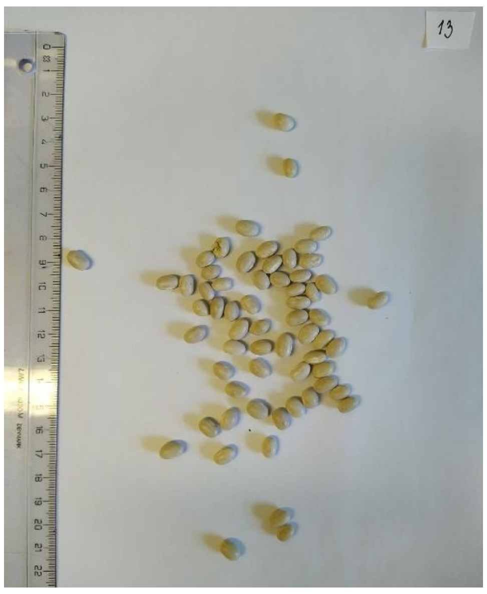

Gramineae family

Barley
Hordeum vulgare L.

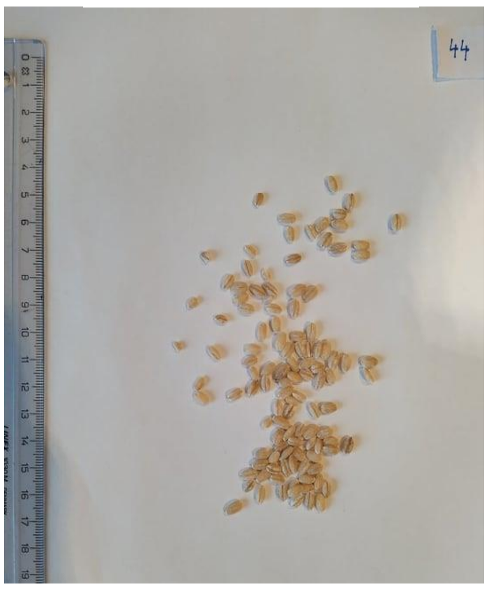

Rice
Oryza sativa L.

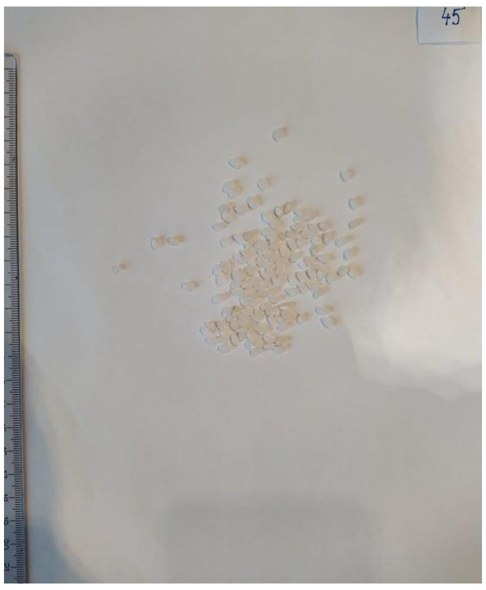

Wheat
Oryza sativa L.

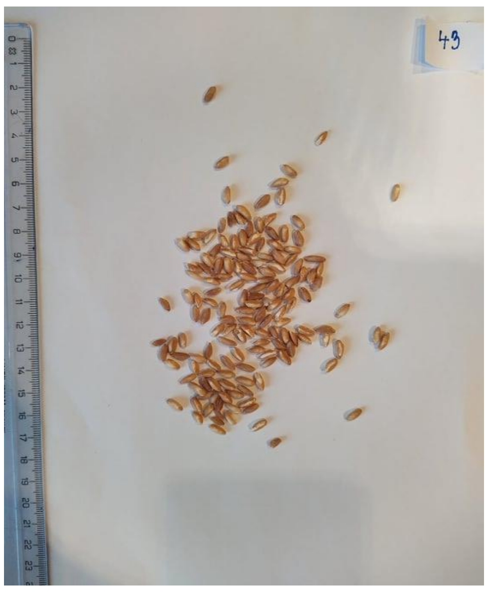

Lamiaceae family

Chia seed
Salvia hispanica L.

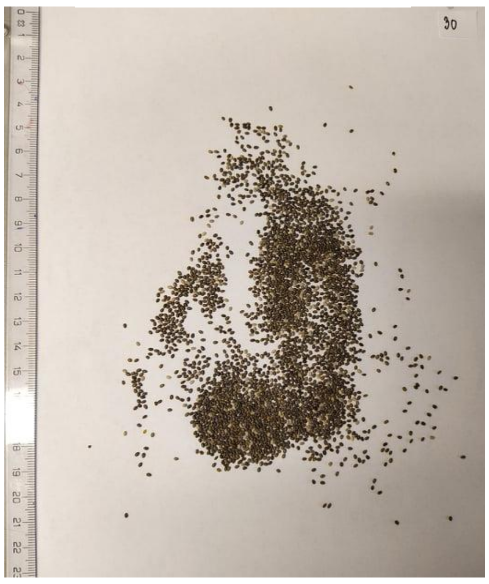

Linaceae family

Linseed
Linum usitatissimum L.

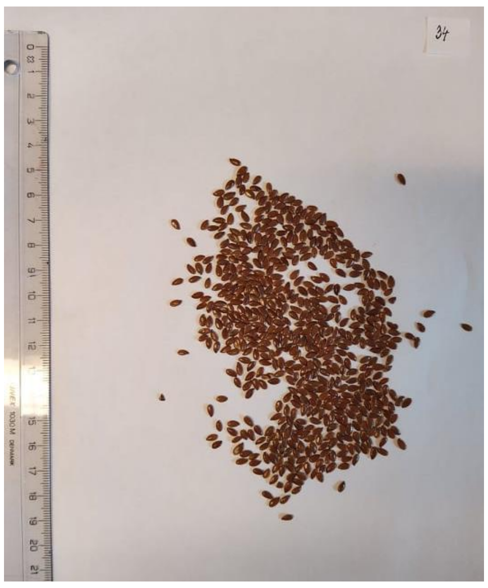

Picture S1 – Sample pictures

Pedaliaceae family

Sesame
Sesamum indicum L.

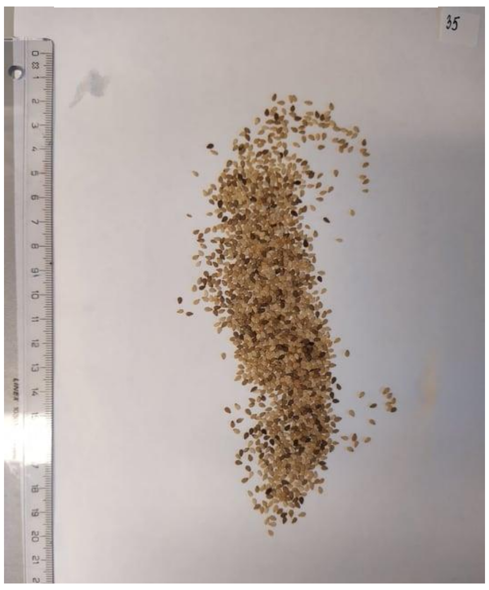

Solanaceae family

Bell pepper, green
Capsicum annuum L.

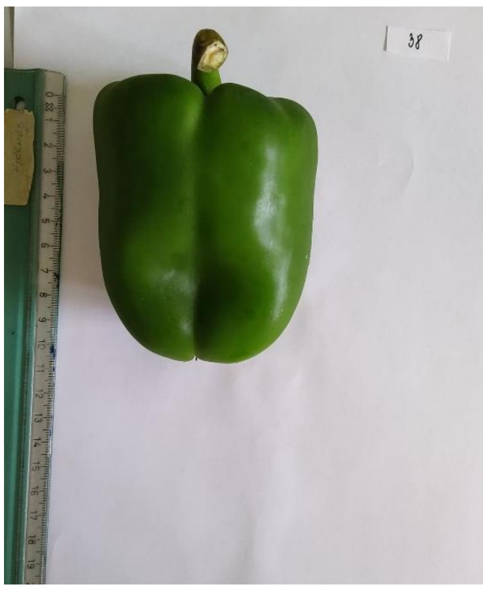

Bell pepper, red
Capsicum annuum L.

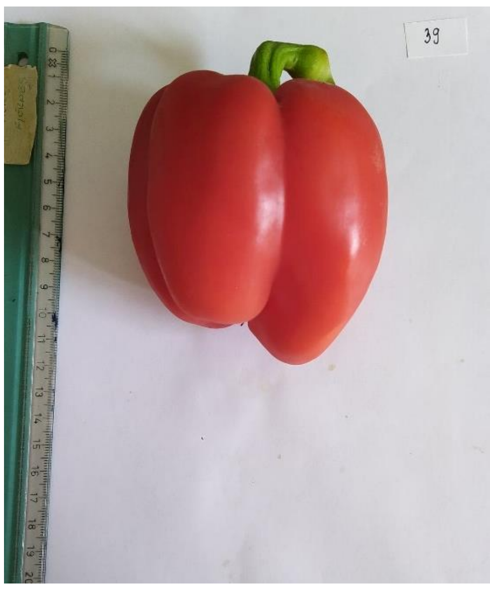

Bell pepper, yellow
Capsicum annuum L.

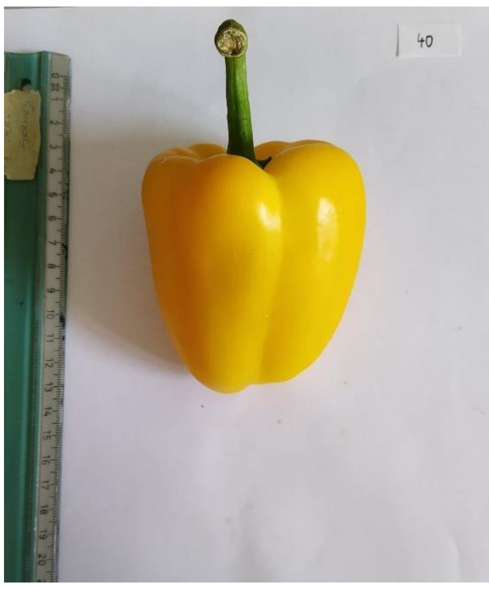

Eggplant
Solanum melongena L.

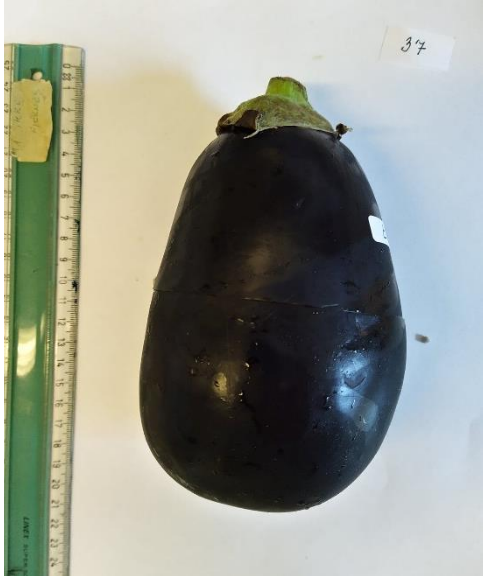

Potato
Solanum tuberosum L.

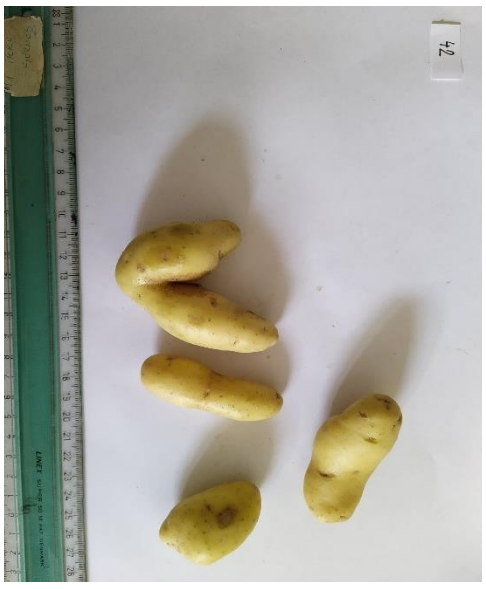

Tomato
Solanum lycopersicum L.

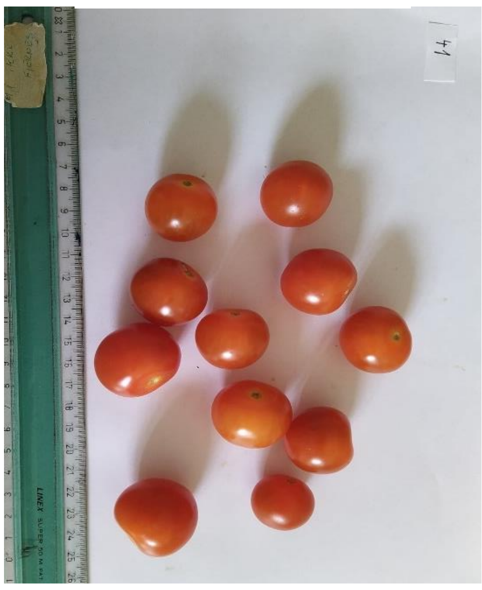

Latin names representing the common English names of samples were retrieved online [34,35].

* This type of bean belongs to *Phaseolus coccineus* L., also known as Runner bean, Scarlet runner bean, or Multiflora bean [36].
