# Supplementary material for: Lectin Activity in Commonly Consumed Plant-Based Foods: Calling for Method Harmonization and Risk Assessment
Source: Foods. 2021 Nov 13;10(11):2796. doi: 10.3390/foods10112796 (PMC8618113; doi:10.3390/foods10112796)
Supplement: Supplementary file 1 [file foods-10-02796-s001.zip › Picture S2 - Differently displayed results.pdf]

Picture S2 – Differently displayed results

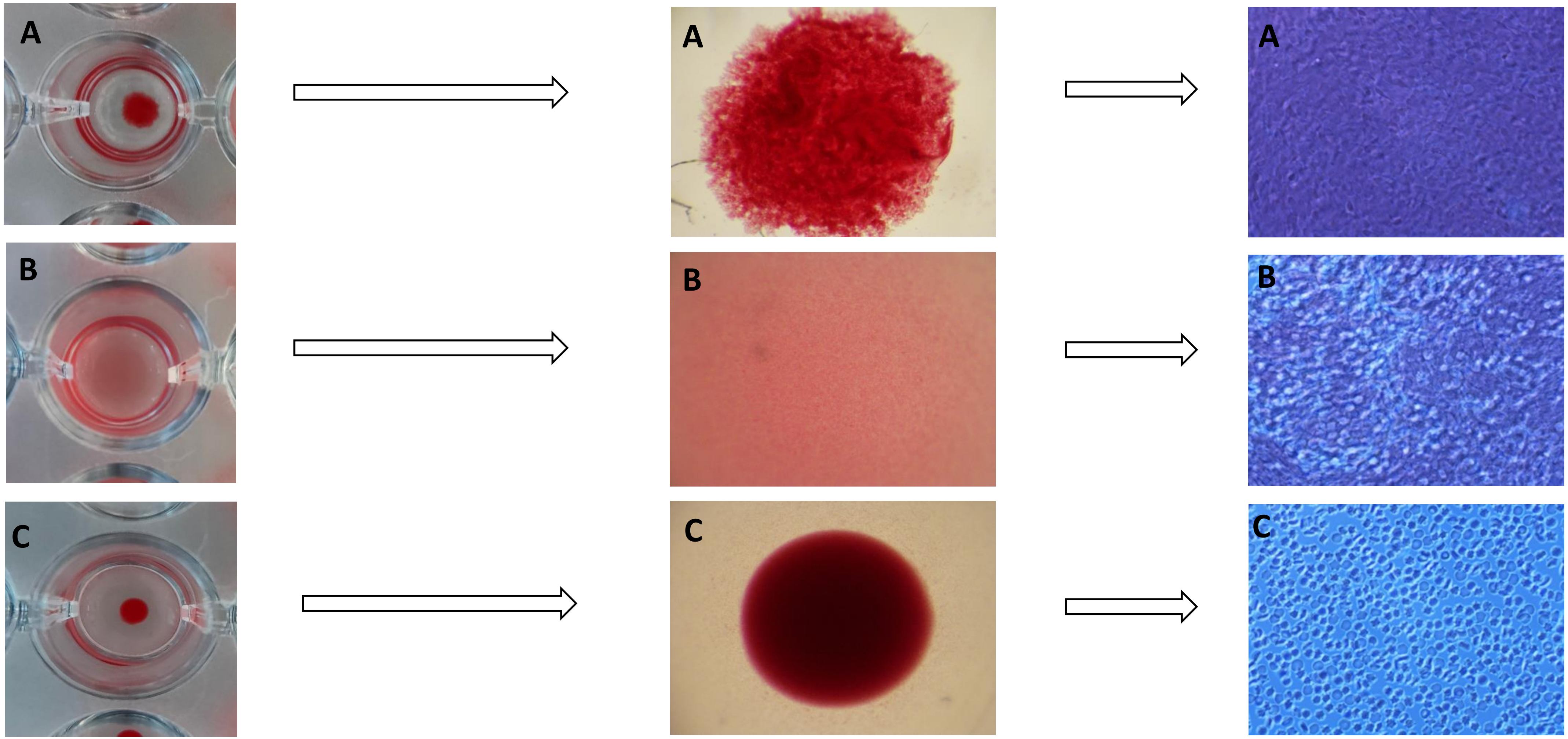

The effect of lectins on rabbit erythrocytes. Rabbit erythrocytes incubated with dilutions of raw kidney bean extracts and subsequently centrifuged. Panel A. A 1/64 dilution that shows an irregular shape. Panel B. A 1/512 dilution that shows agglutination. Panel C. A 1/4096 dilution that shows erythrocytes gathered at the bottom of the well. See paper for experimental details. Recorded with a Realme X2 Pro phone with Ultra Macro mode. 2448 × 3264 pixels, focal length: 2.13 mm.

A 50 X magnification. Panel A. The erythrocytes gather in an irregular manner. Panel B. The erythrocytes are evenly distrubuted in the well. Panel C. The erythrocytes are centered in a regular shape at the bottom of the well. The individual wells are magnified with a Leica MZ 12_5 steriomicroscope.

A 650 X magnification of solutions. 10 µl of solution was placed between a microscope slide and a cover glass. Panel A. Disintegrated erythrocytes and lack of normal cell structure. Panel B. Individual erythrocytes are visible. Panel C. Normal cell structure. Individual erythrocytes are well separated. Magnified with a Leica DM 2000, equipped with a Leica L63x/0.70 objective and a Leica CLS 150X light source. Recorded with a Nikon DS-Fi2.
