# Supplementary material for: Lectin Activity in Commonly Consumed Plant-Based Foods: Calling for Method Harmonization and Risk Assessment
Source: Foods. 2021 Nov 13;10(11):2796. doi: 10.3390/foods10112796 (PMC8618113; doi:10.3390/foods10112796)
Supplement: Supplementary file 1 [file foods-10-02796-s001.zip › Table S1 - Nitrogen results.pdf]

Table S1 – Nitrogen results

Table S1. Nitrogen (N) and protein content in unprocessed samples

**Family**<br>**Common name**	**Species name**[a]	**N [%]**[b]	**Conversion factor**[c]	**Protein [%] ± SD**[d]
**Adoxaceae**				
Elderberry - ripe	*Sambucus nigra* L.	1.1	5.6	6.0 ± 0.02
Elderberry - unripe	*Sambucus nigra* L.	1.3	5.6	7.1 ± 0.02
**Amaranthaceae**				
Nigella seed	*Nigella sativa* L.	3.7	5.3	19.6 ± 0.05
Quinoa, black	*Chenopodium quinoa* Willd.	2.1	5.3	11.3 ± 0.03
Quinoa, red	*Chenopodium quinoa* Willd.	2.2	5.3	11.7 ± 0.03
Quinoa, white	*Chenopodium quinoa* Willd.	2.7	5.3	14.5 ± 0.04
**Cannabaceae**				
Hemp seed	*Cannabis sativa* L.	5.5	5.3	29.3 ± 0.08
**Fabaceae**				
Adzukki bean	*Vigna angularis* L.	3.4	5.4	18.5 ± 0.05
Beluga lentil	*Lens culinaris* L.	3.9	5.4	20.8 ± 0.06
Black bean	*Phaseolus vulgaris* L.	3.8	5.4	20.3 ± 0.05
Borlotti bean	*Phaseolus vulgaris* L.	3.3	5.4	18.0 ± 0.05
Brown lentil	*Lens culinaris* L.	4.1	5.4	22.0 ± 0.06
Chickpea, brown	*Cicer arietinum* L.	3.1	5.4	16.7 ± 0.05
Chickpea, yellow	*Cicer arietinum* L.	3.4	5.4	18.1 ± 0.05
Cowpea	*Vigna unguiculata* L.	3.8	5.4	20.4 ± 0.06
Edamame bean	*Glycine max* L.	3.7	5.5	20.1 ± 0.05
Fava bean	*Vicia faba* L.	4.5	5.4	24.4 ± 0.07
Green bean, broad	*Phaseolus coccineus* L.	3.1	5.4	16.7 ± 0.05
Green bean, haricot vert	*Phaseolus vulgaris* L.	4.0	5.4	21.6 ± 0.06
Green lentil	*Lens culinaris* L.	4.0	5.4	21.6 ± 0.06
Green lentil, le Puy	*Lens culinaris* L.	5.1	5.4	27.3 ± 0.07
Horse bean	*Vicia faba* L.	4.1	5.4	22.2 ± 0.06
Kidney bean	*Phaseolus vulgaris* L.	3.8	5.4	20.5 ± 0.06
Lima bean	*Phaseolus lunatus* L.	3.4	5.4	18.5 ± 0.05
Mung bean	*Vigna radiata* L.	3.9	5.4	20.8 ± 0.06
Pea leave	*Pisum sativum* L.	5.1	5.6	28.5 ± 0.08
Pinto bean	*Phaseolus vulgaris* L.	3.2	5.4	17.4 ± 0.05
Rashti bean	*Phaseolus vulgaris* L.	3.8	5.4	20.5 ± 0.06
Red lentil	*Lens culinaris* L.	4.6	5.4	25.0 ± 0.07
Soybean	*Glycine max* L.	6.5	5.5	35.7 ± 0.10
Sugar pea	*Pisum sativum* L.	3.8	5.4	20.6 ± 0.06
Sugar snap	*Pisum sativum* L.	3.4	5.4	18.1 ± 0.05
Urid bean	*Vigna mungo* L.	3.8	5.4	20.3 ± 0.05
White bean	*Phaseolus vulgaris* L.	3.8	5.4	20.6 ± 0.06
**Gramineae**				
Barley	*Hordeum vulgare* L.	2.0	5.4	10.6 ± 0.03
Rice	*Oryza sativa* L.	1.4	5.4	7.3 ± 0.02
Wheat	*Triticum aestivum* L.	2.1	5.4	11.4 ± 0.03
**Lamiaceae**				
Chia seed	*Salvia hispanica* L.	3.3	5.3	17.3 ± 0.05
**Linaceae**				
Linseed	*Linum usitatissimum* L.	3.7	5.3	19.5 ± 0.05
**Pedaliaceae**				
Sesame	*Sesamum indicum* L.	3.4	5.3	17.9 ± 0.05
**Solanaceae**				
Bell pepper, green	*Capsicum annuum* L.	1.7	5.4	9.2 ± 0.02
Bell pepper, red	*Capsicum annuum* L.	1.4	5.4	7.5 ± 0.02

Table S1 – Nitrogen results

Bell pepper, yellow	*Capsicum annuum* L.	1.1	5.4	6.0 ± 0.02
Eggplant	*Solanum melongena* L.	2.1	5.4	11.2 ± 0.03
Potato	*Solanum tuberosum* L.	1.4	5.4	7.5 ± 0.02
Tomato	*Solanum lycopersicum* L.	2.2	5.4	11.7 ± 0.03

[a] Latin names representing the common English names of samples were retrieved online [34,35].
[b] Results are rounded to the nearest tenths.
[c] Conversion factors were established elsewhere [37,38].
[d] The values (n = 1) ± standard deviation (SD). SD was calculated from the positive control, which was analysed 8 times under reproducible conditions.
